# Revised diagnostic criteria for plasma cell leukemia: results of a Mayo Clinic study with comparison of outcomes to multiple myeloma

**DOI:** 10.1038/s41408-018-0140-1

**Published:** 2018-11-15

**Authors:** Praful Ravi, Shaji K. Kumar, Lindsey Roeker, Wilson Gonsalves, Francis Buadi, Martha Q. Lacy, Ronald S. Go, Angela Dispenzieri, Prashant Kapoor, John A. Lust, David Dingli, Yi Lin, Stephen J. Russell, Nelson Leung, Morie A. Gertz, Robert A. Kyle, P. Leif Bergsagel, S. Vincent Rajkumar

**Affiliations:** 1 Department of Internal Medicine, Mayo Clinic, Rochester, MN USA; 2 Division of Hematology, Mayo Clinic, Rochester, MN USA; 3 Section of Hematologic Oncology, Memorial Sloan Kettering Cancer Center, New York, NY USA; 4 Division of Nephrology and Hypertension, Mayo Clinic, Rochester, MN USA; 5 Division of Hematology and Oncology, Mayo Clinic Arizona, Scottsdale, AZ USA

## Abstract

The current definition of plasma cell leukemia (PCL)— ≥ 20% circulating plasma cells (CPCs) on peripheral smear and plasma cell count ≥ 2 × 10^9^/L—may be too stringent. We reviewed outcomes of 176 multiple myeloma (MM) patients diagnosed between 1971 and 2016, and who had CPCs detectable at diagnosis, to determine whether a lower threshold could be used to diagnose PCL. Median overall survival (mOS) was 1.1 years (95% CI 0.8–1.4) and was similar between patients with < 5% (*n* = 54, mOS = 1.4 years [0.7–2.0]), 5–19% (*n* = 63, mOS = 1.1 years [0.7–1.4]), and ≥ 20% CPCs (*n* = 59, mOS = 1.1 years [0.7–1.5], *p* = 0.349). As survival was similar between those with 5–19% and ≥ 20% CPCs, we stratified patients by < 5% (mOS = 1.4 years [0.7–2.0]) and ≥ 5% CPCs (mOS = 1.1 years [0.8–1.4], *p* = 0.154). Outcomes of those with ≥ 5% CPCs were much poorer when compared with a cohort of MM patients diagnosed between 1971 and 2016, who did not have CPCs at diagnosis (*n* = 9724, mOS = 4.4 yrs [4.3–4.5], *p* < 0.001); survival was also lower in patients diagnosed after 2001 with ≥ 5% CPCs (*n* = 62, mOS = 1.4 years [0.8–2.5]) compared with patients with standard risk (*n* = 1326, mOS = 7.5 years [7.0–8.7]) and high-risk MM (*n* = 381, mOS = 4.3 years [3.5–4.9], *p* < 0.001). We therefore propose that the definition of PCL be revised to patients with ≥ 5% CPCs on peripheral blood smear, who otherwise meet diagnostic criteria for MM.

## Introduction

Plasma cell leukemia (PCL) is an uncommon but aggressive malignancy that accounts for 1–2% of all plasma cell dyscrasias. First described in the early 1900s, PCL has been defined as the presence of ≥ 20% circulating plasma cells (CPCs) on conventional white blood cell differential count and an absolute plasma cell count of ≥ 2 × 10^9^/L in the peripheral blood^1,2^. This definition includes both primary PCL (pPCL)—arising de novo, in the absence of antecedent multiple myeloma (MM)—and secondary PCL (sPCL), which describes leukemic transformation of MM. PCL is clinically and biologically distinct from MM, with a younger age at diagnosis, higher propensity to show visceral and extramedullary involvement, and unique biologic, immunophenotypic, and cytogenetic characterristics^3^.

The current definition of PCL is too restrictive, requiring a very high level of CPCs to be detected on conventional white blood cell differential count, and patients with MM, who have detectable CPCs at a level < 20% threshold, may have similar outcomes to those with ≥ 20% CPCs. Indeed, in 2013, the International Myeloma Working Group called for studies to investigate revision of the diagnostic criteria for PCL^3^. Two such studies have since shown that MM patients with either ≥ 2% or ≥ 5% CPCs, as measured on a peripheral smear, have similar outcomes to patients with pPCL, suggesting that a lower threshold of CPCs be used to define PCL^4,5^.

Based on these considerations, we sought to review our outcomes of MM patients with CPCs detectable on peripheral blood smears at diagnosis and attempt to determine the optimal threshold for the diagnosis of PCL, as well as comparing the outcomes of these patients with patients with MM without CPCs. We hypothesized that MM patients with ≥ 5% CPCs would have similar outcomes to those with traditionally defined PCL, thereby validating earlier studies and providing confirmatory evidence for updating diagnostic criteria for PCL.

## Subjects and methods

After Institutional Review Board approval, all patients who were seen with newly diagnosed MM at our institution between 1 January 1971 and 31 December 2016, and who had CPCs identified on a peripheral blood smear within 30 days of diagnosis were included in this study. Patients in whom a peripheral blood smear was not available before the start of therapy were excluded, as were those who had received previous therapy. The percentage of CPCs was derived from the conventional white blood cell differential count, which consists of a microscopic evaluation of Wright-Giemsa-stained peripheral blood smears. When more than one peripheral smear containing CPCs was available before the start of therapy, the smear with the highest percentage of CPCs was used. Other clinical variables available at the time of diagnosis and information on treatment were abstracted from paper and electronic medical records.

A cohort of MM patients diagnosed at our institution between 1971 and 2016 not meeting the criteria for PCL was identified from our institutional database for comparative analysis. Cytogenetic information was collected from patients who underwent fluorescence in-situ hybridization (FISH) evaluation within 6 months of diagnosis and used to classify these patients as standard risk [trisomies, t(11;14) and t(6;14)] or high-risk MM [t(4;14), t(14;16), t(14;20), and del(17p)].

The primary outcome was overall survival (OS), which was defined as the duration between date of diagnosis and death, or censored at last follow-up. Survival curves were generated using the Kaplan–Meier method and the log-rank tests used to make comparisons between groups. Statistical analysis was performed using SPSS v.22 (IBM Corp., Armonk, NY, USA).

## Results

A total of 176 patients who had CPCs present at the time of MM diagnosis were included in this study (Table 1). Median age at diagnosis was 62 years and the slight majority (56%) of patients were male; median follow-up was 6.8 years (95% confidence interval 4.5–9.0). Median levels of hemoglobin, white blood cell count, and platelets were 9.1 g/dL, 7.4 × 10^9^/L, and 133 × 10^9^/L, respectively. The median number and percentage of CPCs on the peripheral smear at diagnosis was 0.7 × 10^9^/L and 9.8%, respectively, with relatively similar numbers of patients having < 5% CPCs (*n* = 54, 31%), 5–19% CPCs (*n* = 63, 36%), and ≥ 20% CPCs (*n* = 59, 34%).

**Table 1:** Baseline characteristics of MM patients with detectable CPCs on peripheral blood smear at diagnosis, 1971–2016

	*N*
Sex (%)	
Male	99 (56)
Female	77 (44)
Age, median (range)	62 (34–93)
Year of diagnosis (%)	
1971–1980	48 (27)
1981–1990	25 (14)
1991–2000	31 (18)
2001–2010	33 (19)
2011–2016	39 (22)
Hemoglobin, g/dL, median (range)	9.1 (5.0–14.8)
WBC, × 10^9^/L, median (range)	7.4 (0.9–196.3)
Plasma cells, × 10^9^/L, median (range)	0.7 (0.0–163.9)
Platelets, × 10^9^/L, median (range)	133 (14–430)
Calcium, mg/dL, median (range)	9.7 (4.4–16.0)
Creatinine, mg/dL, median (range)	1.8 (0.6–34.0)
CPCs, %, median (range)	9.8 (0.5–96.0)
< 5 (%)	54 (31)
5–19 (%)	63 (36)
≥ 20 (%)	59 (34)
BMPCs, %, median (range)	80 (4–100)

*BMPCs* bone marrow plasma cells, *CPCs* circulating plasma cells, *WBC* white blood cell count

Information on first-line therapy received by patients is shown in Table 2. The majority of patients (58%) received cytotoxic chemotherapy (without a novel agent) upfront, whereas 35% received a novel agent (a proteasome inhibitor, immunomodulatory agent, or both). Just over 20% of patients underwent a hematopoietic stem cell transplant, with the majority of these (87%) undergoing an autologous transplant.

**Table 2:** First-line therapy in MM patients with detectable CPCs on peripheral blood smear at diagnosis

	*N* (%)
Cytotoxic chemotherapy*	102 (58)
PI-based	37 (21)
IMID-based	7 (4)
IMID + PI	17 (10)
Other	6 (3)
No treatment	6 (3)
NA	1 (1)
Hematopoietic stem cell transplant	39 (22)
Autologous	34 (19)
Allogeneic	5 (3)

*This included any cytotoxic agent(s) given in combination, but without a PI or IMID; PI, IMID and IMID + PI indicates these agent(s) given in combination, with or without cytotoxic chemotherapy

*IMID* immunomodulatory agent, *NA* not available, *PI* proteasome inhibitor

**Fig. 1:**
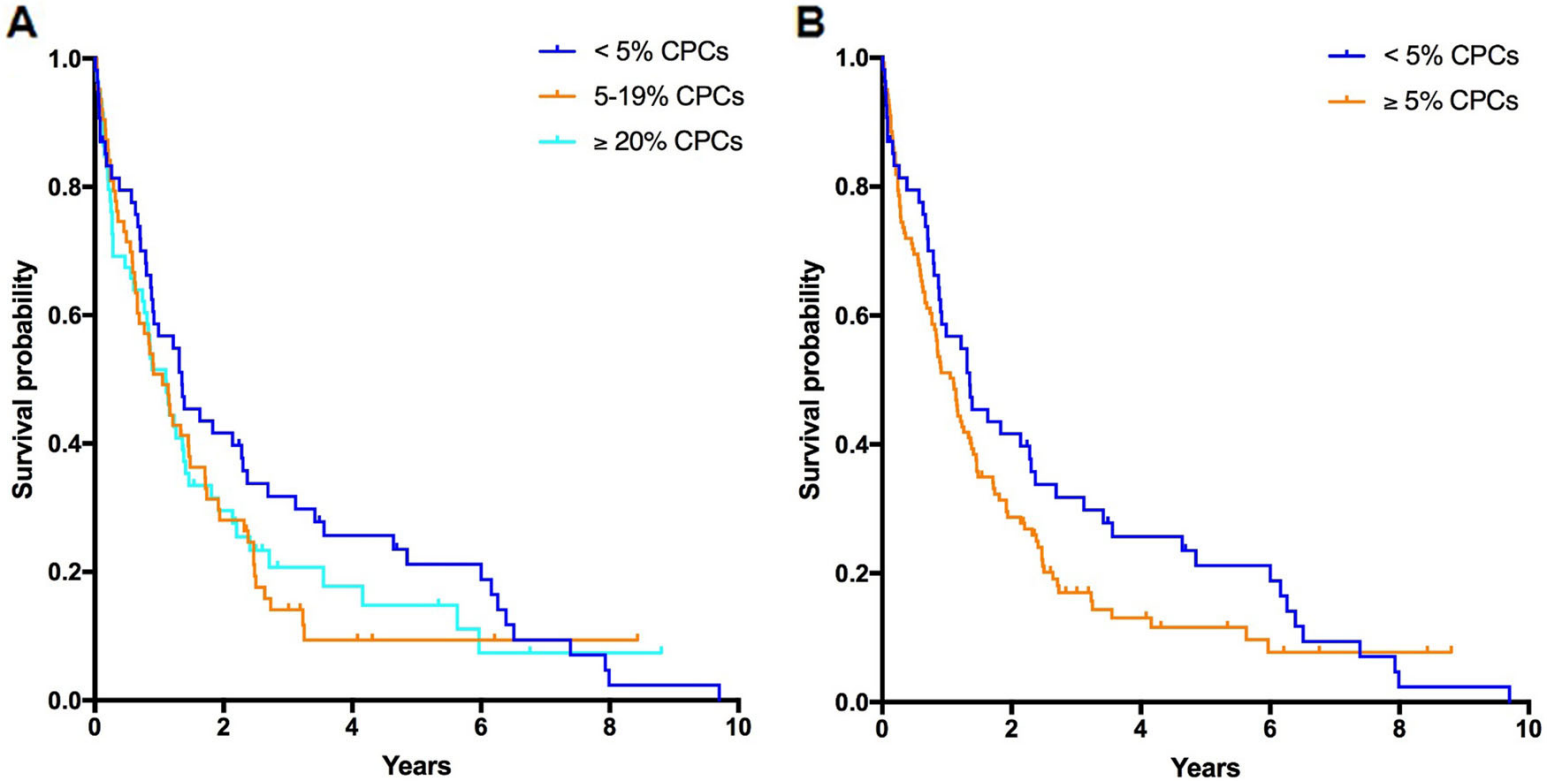
Overall survival in patients with detectable CPCs. Overall survival in patients with detectable CPCs at diagnosis, stratified by < 5%, 5–19%, and ≥ 20% CPCs on peripheral blood smear (**a**), and by < 5% and ≥ 5% CPCs on peripheral blood smear (**b**)

Median OS in the entire cohort was 1.1 years (0.8–1.4). Median OS among patients with < 5%, 5–19%, and ≥ 20% CPCs was 1.4 years (0.7–2.0), 1.1 years (0.7–1.4), and 1.1 years (0.7–1.5, log-rank *p* = 0.349, Fig. 1a), respectively. Due to the similarity in the curves between patients with 5–19% and ≥ 20% CPCs, we proceeded to stratify patients by < 5% CPCs (*n* = 54, median OS: 1.4 years [0.7–2.0] and ≥ 5% CPCs (*n* = 122, median OS: 1.1 years [0.8–1.4], log-rank *p* = 0.154, Fig. 1b). Corresponding 3-year OS rates were 32% (20–44) and 17% (9–25), respectively.

**Fig. 2:**
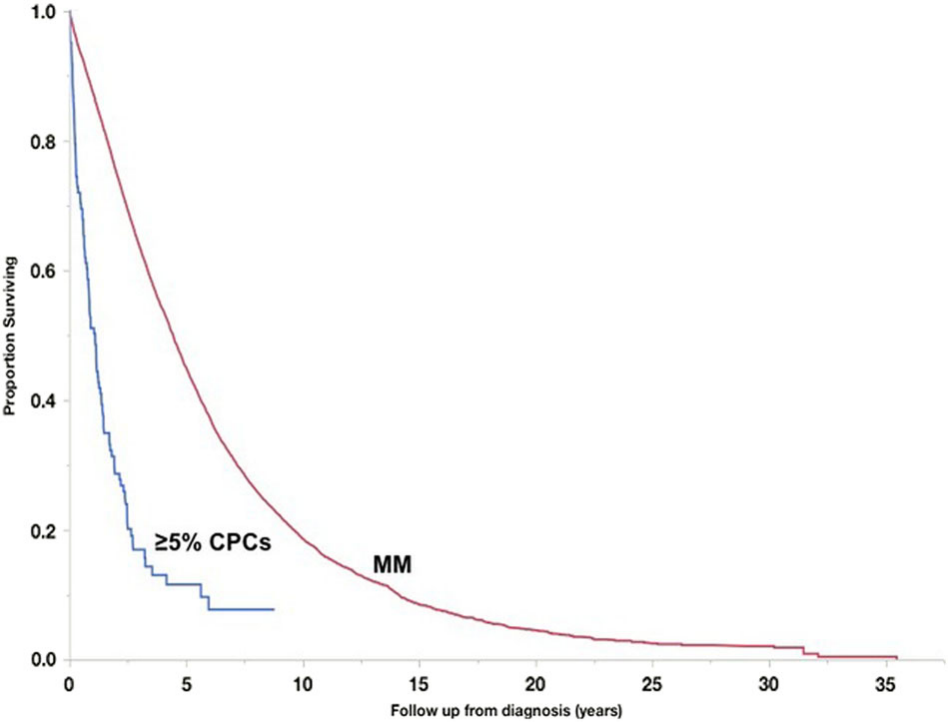
Comparison of overall survival with MM. Overall survival of patients with ≥ 5% CPCs on peripheral blood smear compared with a historical cohort of MM patients without detectable CPCs

**Fig. 3:**
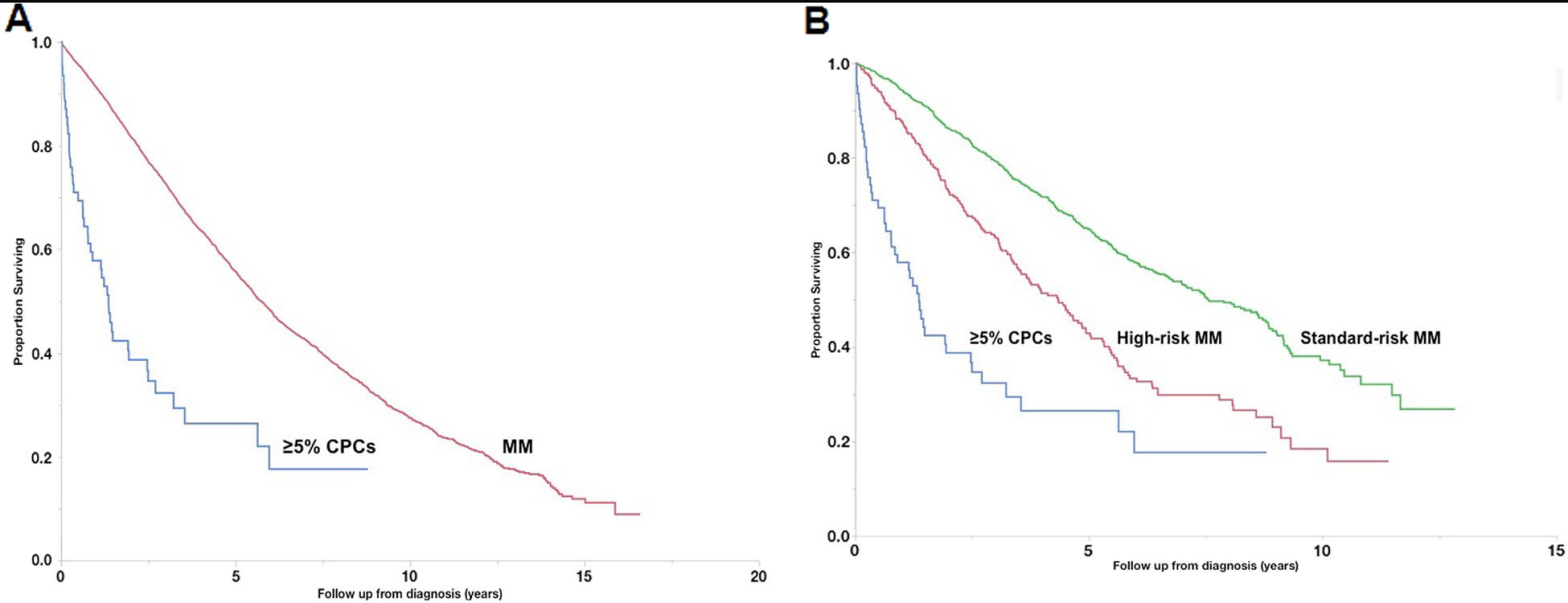
Comparison of overall survival with MM in contemporary era. Overall survival of patients diagnosed from 2001 onwards with ≥ 5% CPCs on peripheral blood smear in comparison with similar cohort of MM patients without detectable CPCs (**a**) and only to MM patients with available cytogenetic information (**b**)

Fig. 2 shows OS of patients with ≥ 5% CPCs (*n* = 122, median OS: 1.1 years [0.8–1.4]) in comparison with a cohort of MM patients diagnosed between 1971 and 2016, and without CPCs at diagnosis (*n* = 9724, median OS: 4.4 years [4.3–4.5], *p* < 0.001). When only considering patients diagnosed after 1 January 2001 (i.e., in the era of novel therapies), survival remained poorer in patients with ≥ 5% CPCs (*n* = 62, median OS: 1.4 years [0.8–2.5]) compared with MM patients (*n* = 5345, median OS: 5.7 years [5.5–6.0], *p* < 0.001, Fig. 3a). We subsequently compared survival of patients with ≥ 5% CPCs (diagnosed from 2001 onwards) with MM patients diagnosed in the same era and in whom cytogenetic information was available (*n* = 1707). Median OS in those with ≥ 5% CPCs (*n* = 62, median OS: 1.4 years [0.8–2.5]) was significantly lower compared with patients with both standard-risk MM (*n* = 1326, median OS: 7.5 years [7.0–8.7]) and high-risk MM (*n* = 381, median OS: 4.3 years [3.5–4.9], *p* < 0.001, Fig. 3b).

## Discussion

The diagnosis of a disease is based upon the uniqueness of its clinical syndrome, presence of particular pathogenetic features and the identification of characteristic histology. For example, with chronic myeloid leukemia, demonstration of the BCR-ABL fusion gene or the Philadelphia chromosome by FISH, PCR, or cytogenetics in the presence of characteristic clinical findings (splenomegaly, leukocytosis) defines the disease^6^. Disease definitions are important, as they enable patients to be treated with specific, and ideally targeted, therapy.

PCL is the most aggressive plasma cell neoplasm and has generally been considered a cousin of MM. Its presentation is notably distinct to MM, with a median age of diagnosis of 55 years (a decade before MM usually presents) and often showing extramedullary involvement, giving rise to hepatosplenomegaly or lymphadenopathy in up to a sixth of cases^7^. Patients have higher levels of β2-microglobulin and lactate dehydrogenase, and lower levels of hemoglobin and albumin in comparison with MM^8^. The disease biology is different, with an increased incidence of hypodiploidy and IgH translocations—typically t(11;14)—and reduced expression of adhesion molecules (such as NCAM and LFA-1)^9^, which may be responsible for the emergence of plasma cells from the bone marrow. Although the treatment of PCL has borrowed much from MM, with bortezomib-based induction therapy followed by autologous and/or allogeneic transplantation in eligible patients^9^, outcomes are often poor, even in the era of novel agents, with median OS in the region of 1 year being seen in population-based data^10,11^.

Given the clinical and biologic distinction of PCL from MM, it is necessary to ensure that its definition is optimized; the traditional definition of PCL ( ≥ 20% CPCs and ≥ 2 × 10^9^/L absolute plasma cells) may be too stringent and inadvertently classify patients as MM when their disease is more aggressive and behaves similar to PCL. In this regard, our study is a valuable addition to the literature, as we noted that survival outcomes for MM patients with any level of CPCs at diagnosis were significantly worse than those without CPCs; moreover, survival was similar between patients with 5–19% and ≥ 20% CPCs at diagnosis. Finally, survival was markedly inferior among those with ≥ 5% CPCs in comparison with patients with high-risk MM, highlighting that these patients truly have a distinct disease phenotype.

Our results corroborate two other studies that have been published in the past few years. A Chinese study reported on 767 patients with newly diagnosed MM in the contemporary era (2004–2012), including 108 with CPCs at the time of diagnosis. They noted that those with ≥ 2% CPCs had worse survival than patients with < 2% CPCs, and that the former had similar survival to 33 patients with traditionally defined pPCL^4^. A multi-center study from Spain evaluated 482 patients with MM, who had peripheral blood smears available at diagnosis between 2008 and 2013, including 100 patients who had CPCs present. They found that patients with 5–20% CPCs had similar or worse survival compared with those with > 20% CPCs (i.e., PCL), whereas survival was similar between those with 1–4% CPCs and no CPCs, although these findings were limited by the relatively small number of patients (*n* = 17) who had ≥ 5% CPCs^5^. Taken together with our results, it is apparent that the disease trajectory in patients with ≥ 5% CPCs is similar to that seen in pPCL (as defined by ≥ 20% CPCs) but notably distinct to that of high-risk MM. Therefore, we propose that the definition of PCL be revised to the presence of ≥ 5% CPCs on a conventional peripheral blood smear.

A surprising finding from our study was the relatively poor survival seen in patients who had < 5% CPCs diagnosis (median OS of 1.4 years) in comparison with that seen among patients with 1–4% CPCs in the Spanish study (median OS of 50 months)^5^. The major reason for this discrepancy is likely due to the large proportion of patients ( > 80%) with < 5% CPCs, who were treated before the era of novel agents (i.e., before 2001), with the majority of these patients receiving melphalan-based combination chemotherapy and only one patient receiving a hematopoietic stem cell transplant. Indeed, our results are similar to population-based data from the SEER database, which showed a median OS of ~6 months for pPCL patients diagnosed before 2001^10^.

To our knowledge, this analysis includes the largest cohort of MM patients with CPCs present at the time of diagnosis and has the longest follow-up. Nevertheless, there are limitations that may hamper the generalizability of our results. First, the data are from a single tertiary center, which may introduce selection or referral bias; however, the proportion of patients with any CPCs (~2%) and with ≥ 20% CPCs (0.6%) is similar to that seen in population-based data, where 0.6% of MM patients had traditionally defined PCL (i.e., ≥ 20% CPCs)^10^, suggesting that our cohort is fairly representative of the typical myeloma population as a whole. In addition, only ~40% of patients were diagnosed in the era of novel therapy (2001 onwards), which contributed to < 50% of patients receiving a novel agent during induction therapy. Furthermore, the majority of patients ( > 75%) did not receive a hematopoietic cell transplant, an approach that has been shown to lead to encouraging outcomes with a 3-year OS of > 60% in pPCL^12^. We also did not have cytogenetic information available in the majority of our PCL cohort and therefore could not assess the interaction between adverse cytogenetics and percentage of CPCs. We did not study the impact of using different cut-offs for absolute plasma cell count (e.g., 0.5 × 10^9^/L) as we feel that using the percentage of CPCs on a peripheral smear is simpler and easier for the practicing clinician. Finally, assessment of CPCs on a conventional cell count and differential is less sensitive than other techniques, such as slide-based immunofluorescence, although it remains the most widely available method.

In summary, we have shown that MM patients with any CPCs present at diagnosis have poorer outcomes to MM patients without CPCs at diagnosis. Moreover, those with 5–19% CPCs have similar survival to those with ≥ 20% CPCs (i.e., traditionally defined PCL), which, together with prior data from other groups^4,5^, provides confirmatory evidence for revising the diagnostic criteria for PCL. We propose that PCL should be defined as the presence of ≥ 5% CPCs on a conventional peripheral blood smear in patients who meet diagnostic criteria for MM. PCL is a clinically and biologically distinct entity from MM, and specifically high-risk MM, and carries a worse prognosis; we hope that this refinement of its diagnostic criteria leads to better identification of this disease and individualized study of these patients within clinical trials.
